# Clinical significance of frequent somatic mutations detected by high-throughput targeted sequencing in archived colorectal cancer samples

**DOI:** 10.1186/s12967-016-0878-9

**Published:** 2016-05-04

**Authors:** Ashraf Dallol, Abdelbaset Buhmeida, Mahmoud Shaheen Al-Ahwal, Jaudah Al-Maghrabi, Osama Bajouh, Shadi Al-Khayyat, Rania Alam, Atlal Abusanad, Rola Turki, Aisha Elaimi, Hani A. Alhadrami, Mohammed Abuzenadah, Huda Banni, Mohammed H. Al-Qahtani, Adel M. Abuzenadah

**Affiliations:** KACST Technology Innovation Center in Personalized Medicine, King Abdulaziz University, P.O. Box 80216, Jeddah, 21589 Kingdom of Saudi Arabia; Center of Excellence in Genomic Medicine Research, King Abdulaziz University, Jeddah, Kingdom of Saudi Arabia; Faculty of Medicine, King Abdulaziz University, Jeddah, Kingdom of Saudi Arabia; Department of Pathology, Faculty of Medicine, King Abdulaziz University, Jeddah, Kingdom of Saudi Arabia; Department of Obstetrics and Gynecology, Faculty of Medicine, King Abdulaziz University, Jeddah, Kingdom of Saudi Arabia; Faculty of Applied Medical Sciences, King Abdulaziz University, Jeddah, Kingdom of Saudi Arabia; Scientific Chair for Colorectal Cancer, King Abdulaziz University, Jeddah, Kingdom of Saudi Arabia

**Keywords:** Colon cancer, Next-generation sequencing, Mutational hotspots, Somatic, Hotspots

## Abstract

**Background:**

Colorectal cancer (CRC) is a heterogeneous disease with different molecular characteristics associated with many variables such as the sites from which the tumors originate or the presence or absence of chromosomal instability. Identification of such variables, particularly mutational hotspots, often carries a significant diagnostic and/or prognostic value that could ultimately affect the therapeutic outcome.

**Methods:**

High-throughput mutational analysis of 99 CRC formalin-fixed and paraffin-embedded (FFPE) cases was performed using the Cancer Hotspots Panel (CHP) v2 on the Ion Torrent™ platform. Correlation with survival and other Clinicopathological parameters was performed using Fisher’s exact test and Kaplan–Meier curve analysis.

**Results:**

Targeted sequencing lead to the identification of frequent mutations in TP53 (65 %), APC (36 %), KRAS (35 %), PIK3CA (19 %), PTEN (13 %), EGFR (11 %), SMAD4 (11 %), and FBXW7 (7 %). Other genes harbored mutations at lower frequency. EGFR mutations were relatively frequent and significantly associated with young age of onset (*p* = 0.028). Additionally, EGFR or PIK3CA mutations were a marker for poor disease-specific survival in our cohort (*p* = 0.009 and *p* = 0.032, respectively). Interestingly, KRAS or PIK3CA mutations were significantly associated with poor disease-specific survival in cases with wild-type TP53 (*p* = 0.001 and *p* = 0.02, respectively).

**Conclusions:**

Frequent EGFR mutations in this cohort as well as the differential prognostic potential of KRAS and PIK3CA in the presence or absence of detectable TP53 mutations may serve as novel prognostic tools for CRC in patients from the Kingdom of Saudi Arabia. Such findings could help in the clinical decision-making regarding therapeutic intervention for individual patients and provide better diagnosis or prognosis in this locality.

## Background

Colorectal cancer (CRC) is major cause of morbidity and mortality around the world being the third most common cancer type worldwide [1]. Localized statistics show that the age-standardized incidence rates (per 100,000) of CRC in KSA vary between 9.5 in females to 14.1 in males being the most common cancer type in Saudi males [1]. CRC is a heterogeneous disease affected by genetic and epigenetic variations acting as passengers or drivers of the tumor. However, common genetic features of CRC have emerged including mutations affecting APC [2], activating mutations of KRAS or BRAF oncogenes [3], deletions of the 18q [4] and 17p [5] chromosomal regions, microsatellite instability (MSI) [6] with deleterious mutations affecting the tumor suppressor genes TP53 [7]. In terms of methylation, the CpG Island Methylator Phenotype (CIMP) pathway is the second most common pathway in sporadic CRC [8].

In terms of the application of precision medicine and personalized oncology, it is important to identify underlying variations as individually or in combination as such understanding can potentially affect treatment. For example, CRC tumors with high levels of chromosomal instability have a poor prognosis, especially if they are in stage II or III [9]. Conversely, tumors with high microsatellite instability have a better clinical outcome compared to microsatellite-stable tumors [6]. CIMP-positive CRC tumors are usually associated with the proximal colon of older females and often accompanied by BRAF mutations [10]. Male CRC patients who are CIMP negative and carry a polycomb target genes methylation signature have a favorable prognosis [11]. In terms of genetic mutations, KRAS mutations adversely affect patients’ response with anti-EGFR treatment modalities [3]. Furthermore, mutations in the EGFR itself may cause unpredictable responses to such treatments [3]. Mutations in the PIK3CA or BRAF downstream of EGFR signaling may also adversely affect treatment response [3].

We have used the cancer hotspot panel version 2 from Life Technologies in combination with the Ion Torrent personal genome platform in order to investigate the mutational status of 2800 COSMIC (catalogue of somatic mutations in cancer) mutations in 50 oncogenes and tumor suppressor genes in a cohort of CRC cases from Saudi Arabia. The results obtained will help us understand the genetic background of CRC from this population and help implement relevant modalities of precision medicine to the treatment of this disease.

## Methods

### Patients

The material of the present study consist of a series of 99 CRC specimens, retrospectively collected from the archives of Anatomical Pathology Laboratory in King Abdulaziz University (KAUH) and King Faisal Specialist Hospitals (KFSHRC), Jeddah, Kingdom of Saudi Arabia, covering the period from January 2005 to December 2014. Serial sections were cut from paraffin blocks, stained with Hematoxylin and Eosin for routine histological examination, classification, grading and staging following the AJCC staging system [11]. The pertinent clinicopathological data (gender, age, grade, and lymph node status), and follow-up results were retrieved from the patients’ records after obtaining the relevant ethical approvals. DNA was extracted from 10 μm-thin formalin-fixed paraffin-embedded slices using the Qiagen QIAMP Formalin-fixed Parafin-embedded Tissue DNA extraction kit, following the manufacturer’s guidelines.

### Ion PGM library preparation and sequencing

Approximately 10 ng of DNA from each sample, as determined by the Qubit assay (Life Technologies) were used to construct barcoded Ion Torrent adaptor-ligated libraries utilizing the Ion Ampliseq Library Kit 2.0 (Life Technologies) following the manufacturer’s protocol. The cancer genes were amplified in 207 amplicons using the primer pool from the Cancer Hotspot Panel 2.0 (Life Technologies). Templated spheres were prepared using 100 pM of DNA from each library using the Ion OneTouch 2.0 machine. Template-positive spheres from the barcoded libraries were multiplexed and loaded onto Ion chips 316 version 2.0 and sequencing was performed using the Ion Sequencing 200 v2 kit from Life Technologies.

### Variant calling

Processing of the PGM runs was achieved with the Torrent Suite version 4.4.3. The Coverage information, identification of low frequency variants, as well as variant annotation was achieved by the Ampliseq CHPv2 single sample workflow within the Ion Reporter suite v4.6. Somatic mutations with a coverage ≥100 and *p* value of ≤0.05 were included. Variants with near 50/50 distribution of coverage were presumed germ line and excluded from further analysis. In order to increase accuracy of variant calling, variants not previously reported either in dbSNP or COSMIC databases were excluded from further analysis.

### Statistical analysis

All statistical tests were performed using IBM SPSS Statistics version 19. Fisher’s exact test was used to identify statistical significance of correlation between mutational events and clinicopathological factors. The primary endpoints of the study included disease-specific survival (DSS) calculated from the date of diagnosis to the last recorded date of being alive or death caused by CRC. In calculating DSS, patients who died of other or unknown causes were excluded. All survival times were calculated by univariate Kaplan–Meier analysis, and equality of the survival functions between the strata was tested by log-rank (Mantel-Cox) test. Multivariate Cox regression analysis was performed to disclose independent predictors of DSS. All tests were two-sided, and *p*-values <0.05 were considered statistically significant.

## Results

**Fig. 1:**
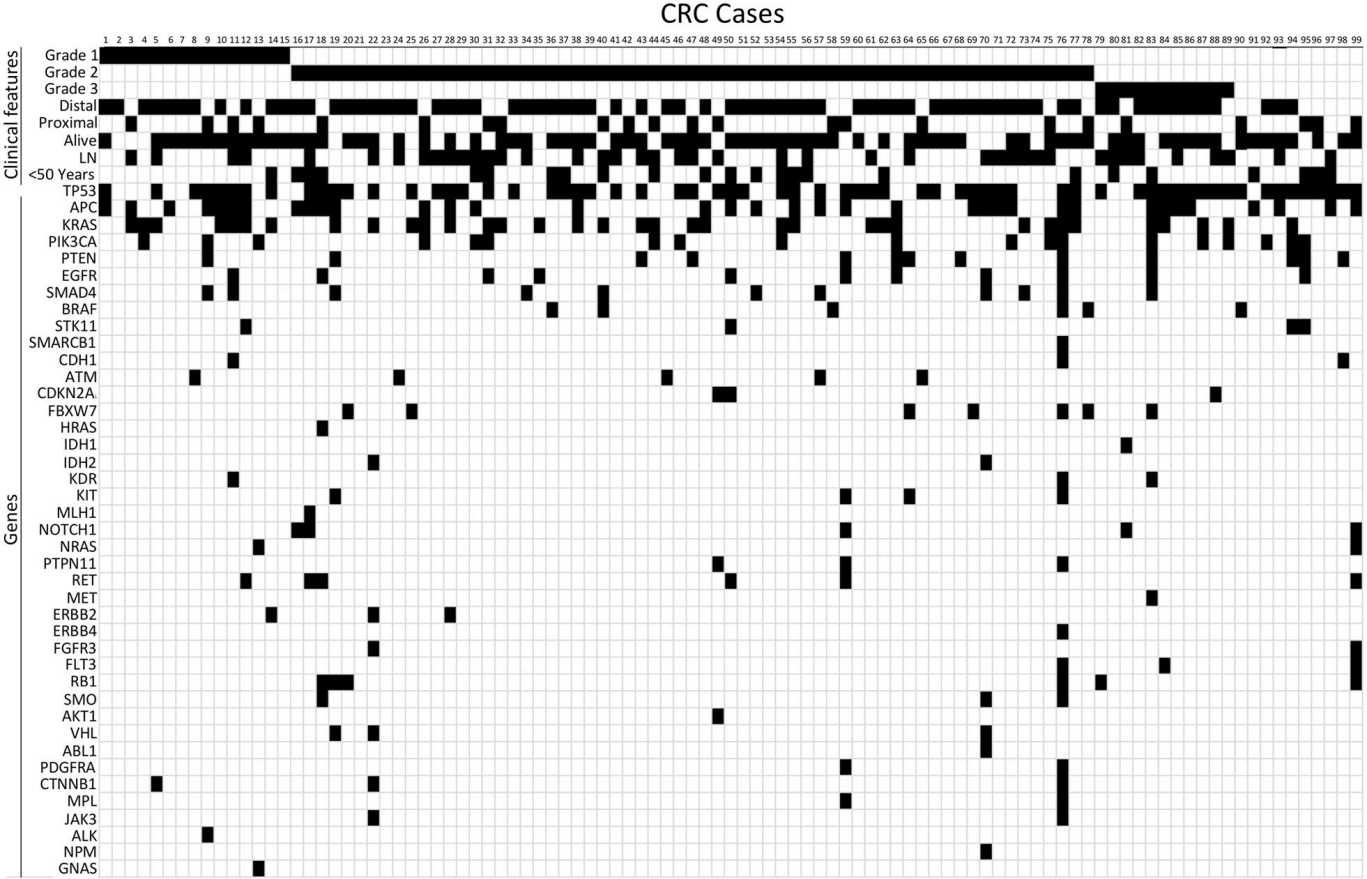
Distribution of the somatic mutations identified in relations to age, tumor location, lymph node metastasis (LN) or tumor grade. A positive value is indicated with a *black square* while a negative value is indicated by a *white square*

**Fig. 2:**
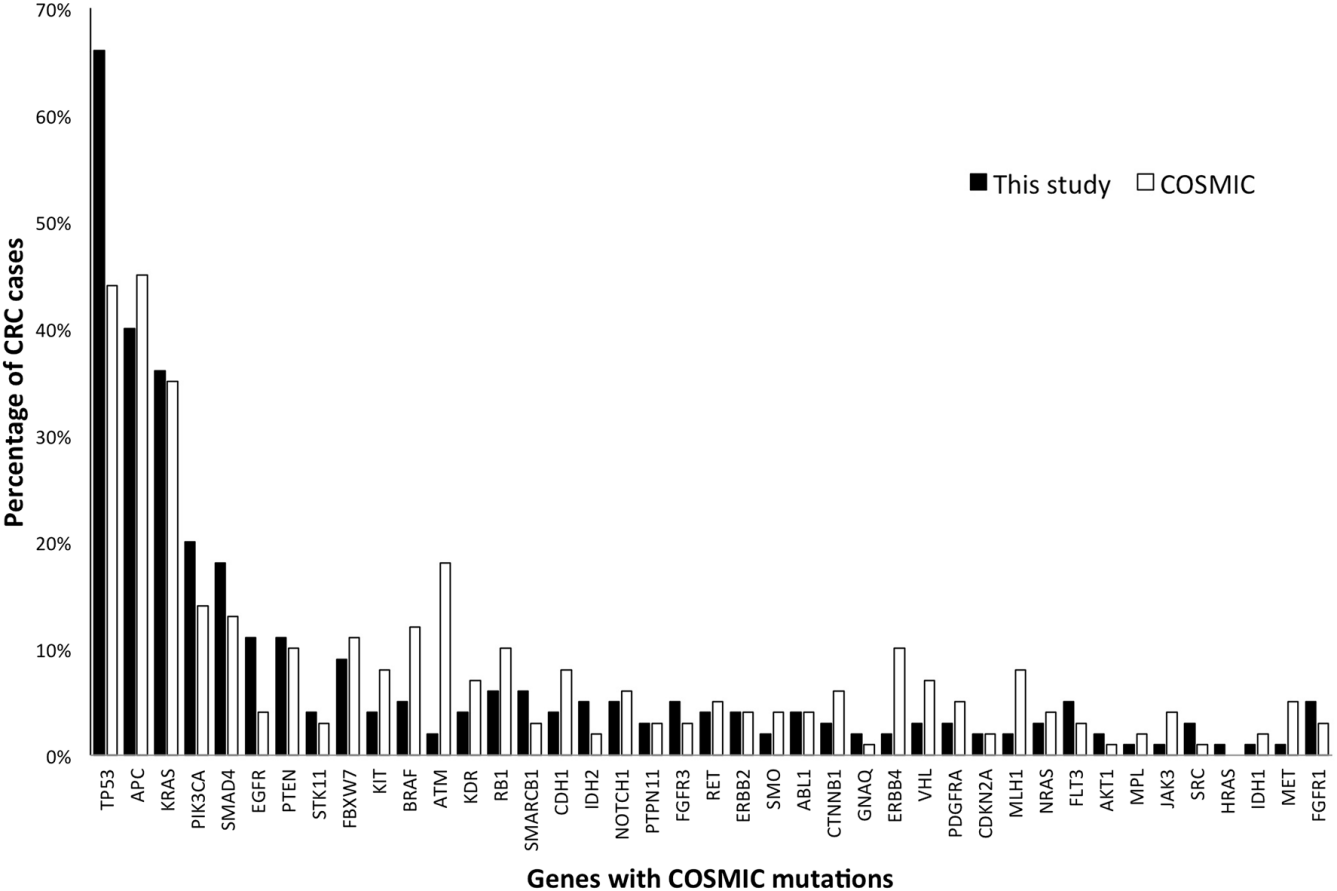
Comparison of the mutation frequency of cancer genes (*x-axis*) as reported in the COSMIC database [12] (*white bars*) and identified in our CRC cohort (*black bars*)

The cancer hotspot panel v2 based on the Ampliseq technology and the ion torrent PGM was utilized to screen 99 archival FFPE samples obtained from colorectal cancer patients diagnosed and treated at KAUH and KFSHRC between 2005-2014. (Table 1) Variants identified were filtered based on coverage level above 100× and *p*-value of <0.05 followed by the exclusion of common variants. Hotspot mutations were identified in 88/99 cases occurring in 41 genes at variable frequency (Table 2). Frequent mutations were identified in TP53 (65 %), APC (36 %), KRAS (35 %), PIK3CA (19 %), SMAD4 (11 %), EGFR (11 %), PTEN (13 %), and FBXW7 (7 %). Less frequent mutations were additionally identified in 33 other genes at a frequency ranging from 1 to 6 % (Fig. 1). In comparison to the mutation frequencies reported by the COSMIC database of mutations detected in cancers originating in the large intestine TP53 and EGFR mutation frequencies are high in our cohort. On the other hand, ATM and ERBB4 mutations are relatively rare. The remaining genes are mutated at a frequency similar to COSMIC reports (Fig. 2).

**Table 1:** Clinical features of 99 CRC patients included in this study

	Number of cases (%)
Number of cases	99
Males	58 (58.6)
Females	41 (41.4)
Age	
Below 50 years old	25 (25.3)
Above 50 years old	74 (74.7)
Tumor location	
Right colon	22 (22.2)
Left colon	47 (47.5)
Rectum	27 (27.3)
Undetermined	3 (03.0)
Lymph node status	
LN+	46 (46.5)
LN−	41 (41.4)
Undetermined	12 (12.1)
Grade	
Grade 1 (well-differentiated)	16 (16.2)
Grade 2 (moderately-differentiated)	61 (61.6)
Grade 3 (poorly-differentiated)	10 (10.1)
Undetermined	12 (12.1)
Survival status	
Recurrence	42 (42.4)
Dead	24 (24.2)
Alive	74 (74.7)
Undetermined	01 (01.0)

**Table 2:** Somatic mutations detected by the cancer hotspot panel v2 in CRC

Gene	Mutations detected
TP53	p.Ala69Val, p.Pro72Ala, p.Pro72Ser, p.Thr81Ile, p.Pro82Leu, p.Ala84Val, p.Pro87Leu, p.Trp91Ter, p.Ser94Ter, p.Ser95Phe, p.Ser96Phe, p.Pro98Leu, p.Ser99Phe, p.Gln100Ter, p.Arg110Leu, p.Leu130Ile, p.Lys132Arg, p.Cys135Ter, p.Leu137Gln, p.Pro151Thr, p.Pro152Leu, p.Gly154Asp, p.Gly154Ser, p.Arg156His, p.Val157Ile, p.Val157Phe, p.Ala161Thr, p.Tyr163Cys, p.Gln165Ter, p.Ser166Leu, p.His168Pro, p.His168Tyr, p.Glu171Lys, p.Val172Ile, p.Val173Ala, p.Val173Met, p.Arg175His, p.Arg175Leu, p.Pro177Ser, p.His179Arg, p.His179Tyr, p.Cys182Tyr, p.Ser183Leu, p.Gly187Ser, p.Pro190 fs, p.Arg196Ter, p.Gly199Glu, p.Arg202Cys, p.Arg209 fs, p.Thr211Ile, p.Arg213Ter, p.Ser215Asn, p.Val216Met, p.Tyr220Asn, p.Tyr220Cys, p.Glu221Gly, p.Gly226Ser, p.Cys229Tyr, p.Tyr234Cys, p.Cys238Tyr, p.Ser240Arg, p.Ser240Asn, p.Gly244Asp, p.Gly245Ser, p.Met246Val, p.Arg248Gln, p.Arg248Trp, p.Arg249Ser, p.Thr253Ile, p.Glu258Gln, p.Gly266Arg, p.Gly266Glu, p.Arg267Gln, p.Ser269Asn, p.Glu271Lys, p.Val272Met, p.Arg273Cys, p.Arg273His, p.Cys275Phe, p.Cys277Phe, p.Gly279Glu, p.Arg280Lys, p.Asp281Asn, p.Arg282Trp, p.Glu285Lys, p.Glu287Lys, p.Arg290His, p.Pro300Leu, p.Pro300Ser, p.Gly302Glu, p.Arg306Ter, p.Glu336Lys, c.559 + 3G > C, c.560-1G > C, c.376-1G > A
APC	p.Asn869Thr, p.Arg876Ter, p.Arg1114Ter, p.Glu1286Ter, p.Ala1296 fs, p.Ala1299Val, p.Ile1307Lys, p.Ile1307 fs, p.Glu1309Ter, p.Glu1309 fs, p.Ala1351Thr, p.Ser1356Ter, p.Ser1360Tyr, p.Gln1367Ter, p.Glu1374Ter, p.Gln1378Ter, p.Glu1379Ter, p.Pro1439Leu, p.Arg1450Ter, p.Glu1461Lys, p.Ser1465 fs, p.Leu1488 fs, p.Leu1488Ter, p.His1490Leu, p.His1490 fs, p.Ser1495 fs, p.Ser1501 fs, p.Thr1556 fs, p.Glu1576Lys, p.Glu1577Ter
KRAS	p.Ala11Val, p.Gly12Val, p.Gly12Ser, p.Gly12Asp, p.Gly13Asp, p.Val14Ile, p.Gln22Lys, p.Gln61His, p.Ala146Thr
PIK3CA	p.Arg88Gln, p.Arg108His, p.Leu339Ile, p.Asn345Lys, p.Asp350Gly, p.Cys420Arg, p.Pro539His, p.Glu542Lys, p.Glu545Asp, p.Glu545Gly, p.Glu545Lys, p.Gln546His, p.Arg1023Ter, p.Thr1025Ala, p.Asn1044Ser, p.His1047Arg, p.His1048Arg, p.Ala1066Thr, p.Ter1069 fs
PTEN	p.Lys6Glu, p.Glu7Ter, p.Asp24Asn, p.Gln110Ter, p.Asp115Asn, p.Cys124Tyr, p.Ala126Val, p.Arg130Gln, p.Gly165Glu, p.Ser170Asn, p.Gln171Ter, p.Arg173Cys, p.Pro246Leu, p.Val255Ile, p.Glu256Lys, p.Pro339Ser
SMAD4	p.Arg100Met, p.Arg135Ter, p.Gln245Ter, p.Gln248Ter, p.Thr259Ile, p.Glu330Lys, p.Ser343Ter, p.Gly352Arg, p.Arg361His, p.Arg361His, p.Arg361His, p.Cys499Tyr, p.Arg531Gln
EGFR	p.Gly109Glu, p.Gly696Arg, p.Pro699Ser, p.Gly719Ser, p.Gly719Cys, p.Gly721Ser, p.Trp731Ter, p.Glu746Lys, p.Thr751Ile, p.Ala755Thr, p.Glu758Lys, p.Leu858Met, p.Gly863Asp, p.Ala864Val, p.Gly873Glu
FBXW7	p.Arg465Cys, p.Arg465His, p.Met467Ile, p.Arg479Gln, p.Arg479Pro, p.Arg479Ter
BRAF	p.Gly469Val, p.Gly469Ala, p.Val600Glu, p.Lys601Glu
RB1	p.Ile680Thr, p.Leu683Phe
RET	p.Cys618Tyr, p.Asp627Gly, p.Glu884Val, p.Glu901Lys, p.Leu923Phe
ATM	p.Val410Ala, p.Phe858Leu, p.Thr1735 fs, p.Leu2866Val
NOTCH1	p.Arg1598His, p.His1601Leu, p.Pro2438Ser, p.Gln2440Ter
STK11	p.Glu165Lys, p.Gly171Ser, p.Gly279Arg, p.Phe354Leu
KIT	p.Pro37Ser, p.Arg49His, p.Asp52Asn, p.Asp572Asn
KDR	p.Lys270Asn, p.Gly1145Glu, p.Pro1354Ser
CDH1	pThr342Ile, p.Thr399Ile
PTPN11	p.Glu69Lys, p.Thr73Ile, p.Glu76Lys
ERBB2	p.Met774Ile, p.Val851Met, p.Thr862Ala, p.His878Tyr
SMO	p.Val404Met, p.Leu412Phe, p.Pro634Leu
CTNNB1	p.Met12Ile, p.Pro16Ser, p.Val22Ile, p.Ser37Phe
VHL	p.Gln132Ter, p.Arg161Gln, p.Arg167Gln, c.341-1G > A
CDKN2A	p.Glu88Lys, p.Arg99Trp, p.Asp125Asn, p.Arg128Gln
FLT3	p.Ser446Leu, p.Lys602Arg, p.Trp603Ter
IDH2	p.Arg140Gln, p.Arg140Trp
FGFR3	p.Asp643Asn, p.Ala719Thr
MPL	p.Ala506Thr, p.Ala519Val, p.Ala519Thr
PDGFRA	p.Pro553Leu, p.Glu563Lys
NRAS	p.Gln61Arg
MET	c.2942-20_2943del22, p.Asn375Ser
MLH1	p.His381Arg
HRAS	p.Glu63Lys
JAK3	p.Ala573Val
AKT1	p.Glu49Lys
ERBB4	p.Pro616Ser
JAK3	p.Ala572Thr
SMARCB1	p.Arg190Gln
ABL1	p.Thr334Ile

Ninety-five different TP53 mutations were detected in 64 patients (Fig. 1; Table 2) with the most common mutations affecting arginine residues 175 (6 cases; p.Arg175His and p.Arg175Leu), 248 (6 cases; p.Arg248Glu and p.Arg248Trp), 273 (5 cases; p.Arg273Cys and p.Arg273His). Furthermore, TP53 mutations are largely concentrated in the DNA binding domain, but mutations affecting the other domains of the protein were also identified at a lesser frequency. Thirty mutations were found affecting the APC gene in 39 patients. APC mutations are not concentrated in a particular domain, however, 21/30 mutations were truncating mutations (Fig. 1; Table 2). The most recurrent APC mutation is affecting the arginine 1450 residue (9 cases; p.Arg1450Ter). Nine different mutations were identified in KRAS (Fig. 1; Table 2) with the most common affecting glycine 12 residue (20 cases; p.Gly12Asp/Ser/Val) followed by changes to the glycine 13 residue recurring 7 times (p.Gly13Asp). The p.Ala146Thr mutation was identified in 5 patients while the p.Gln61His change was identified in two patients. Known pathogenic mutations affecting SMAD4 were found in 6 patients (Fig. 1; Table 2). Overall there were eleven cases with somatic SMAD4 mutations identified in this cohort with the most frequent variant is the p.Arg361His missense mutation in occurring in 3 patients. PIK3CA mutations were identified affecting 19 patients. These mutations were largely occurring in Exon 9 and exon 20 of the protein (13/19 mutations; Table 2) with changes at the glutamic residues 542 and 545 being the most frequent (6/19). Exon 20 mutations occurred in 7/19 cases. Fifteen mutations were identified affecting EGFR in 11 patients (Fig. 1 and Table 2). One mutations was identified in the extracellular receptor L domain (p.Gly109Glu) and 14/15 mutations in the intracellular protein tyrosine kinase domain. p.Glu746Lys occurred 4 times and the p.Gly719Cys/Ser occurring 3 times in our cohort. PTEN mutations were identified in 13 patients with the most common being p.Arg130Gln, p.Asp115Asn and p.Asp24Asn. The remaining mutations identified are summarized in Table 2.

The presence of APC mutations correlated with mutations affecting the EGFR and SMAD4 genes (Pearson’s correlation; *p* = 0.016 and *p* = 0.002, respectively). Similar correlation is also found with SMAD4 and EGFR mutations (*p* = 0.001). Additionally, there is a positive correlation between KRAS and PIK3CA mutations (*p* = 0.004). Positive correlation was also found between PIK3CA and EGFR mutations (*p* = 0.019) as well as PIK3CA and PTEN mutations (*p* = 0.008). The presence of PTEN mutations correlated positively with the presence of SMAD4 mutations (*p* = 0.015), EGFR mutations (*p* = 0.001) as well as FBXW7 mutations *(p* = 0.015). Furthermore, FBXW7 mutations correlated positively with BRAF mutations (*p* = 0.009). In terms of association with clinicopathological parameters, EGFR mutations were significantly associated with young age of onset (Fisher’s exact t-test; *p* = 0.028). Mutations affecting BRAF are associated with tumors arising in the right colon (*p* = 0.023).

**Fig. 3:**
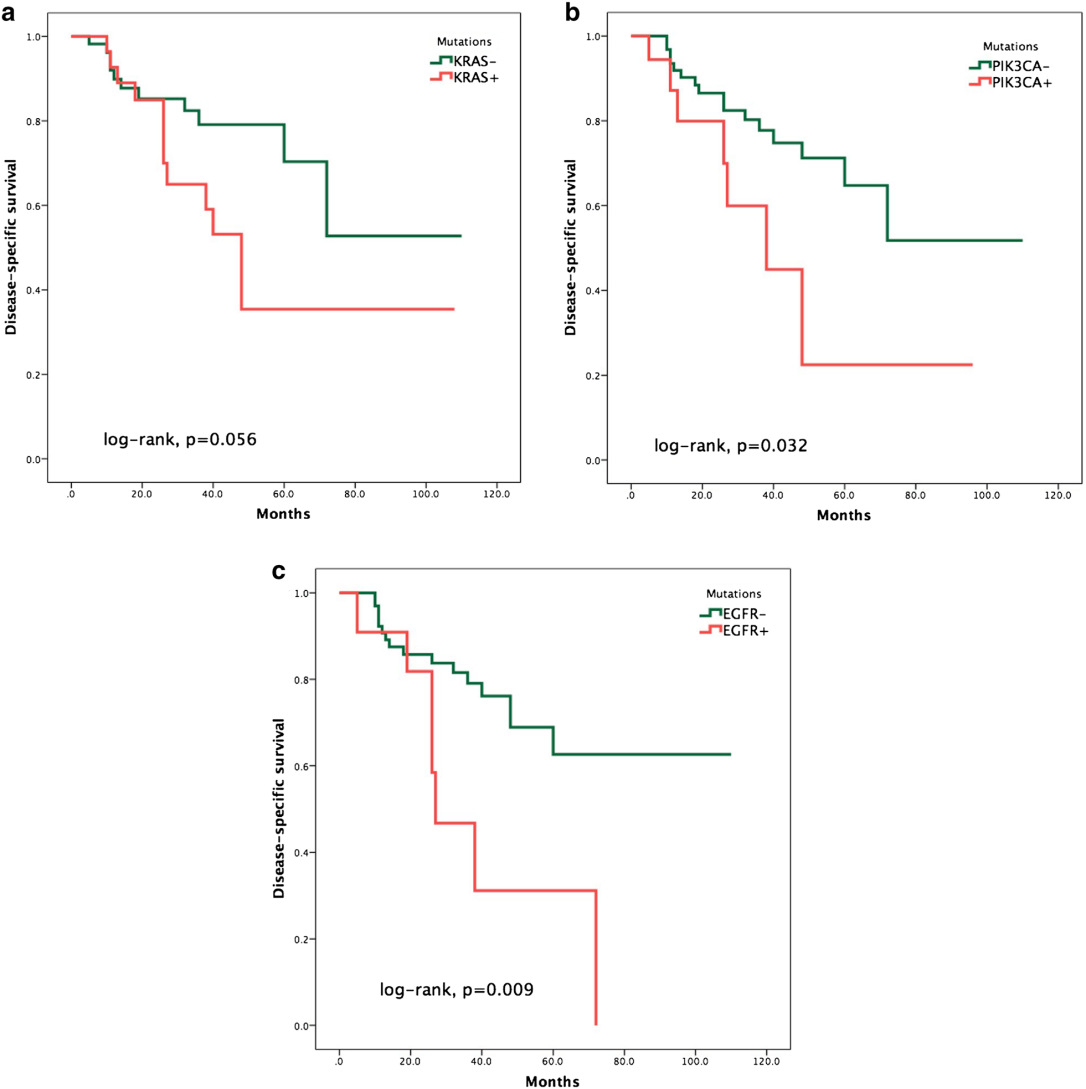
Kaplan-Meier survival curves showing the effects of the presence of somatic mutations in KRAS (**a**), PIK3CA (**b**) and EGFR(**c**) (indicated by “+” sign) on disease-specific survival

**Fig. 4:**
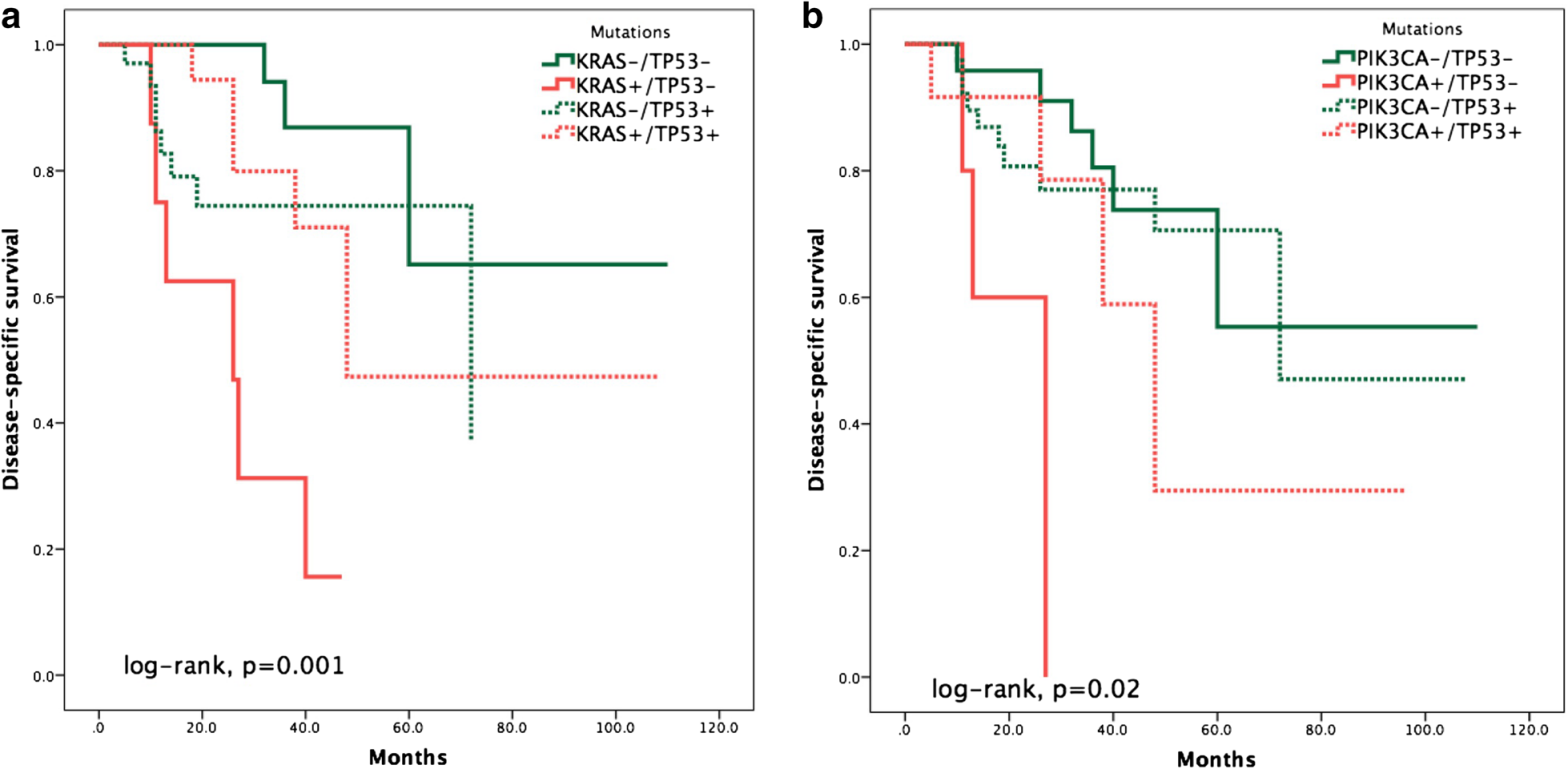
Kaplan-Meier survival curves demonstrating the effects of the presence of somatic mutations in KRAS in the presence or absense of TP53 mutations (**a**), or PIK3CA in the presence or absense of TP53 (**b**) (indicated by “+” sign) on disease-specific survival

In terms of disease-specific survival (DSS), CRC tumors harboring KRAS mutations have shorter DSS prognosis (Kaplan–Meier log rank test, *p* = 0.056; Fig. 3a). However, such prognosis is worsened if the patient has KRAS mutations coupled with wild-type TP53 (Kaplan–Meier log rank test, *p* = 0.001; Fig. 4a). Similarly, PIK3CA mutations are associated with shorter DSS (Kaplan–Meier log rank test, *p* = 0.032; Fig. 3b). However, the effect of PIK3CA mutations on DSS is increased in the background of wild-type TP53 (Fig. 4b). Furthermore, EGFR mutations are associated with significantly shorter DSS in CRC (Kaplan–Meier log rank test, *p* = 0.009; Fig. 3c). Cox’s regression analysis of disease-specific survival indicates that detection of EGFR mutations is an independent marker for poor prognosis in CRC with a hazard ratio of 3.639 (Table 3; *p* = 0.02, CI = 1.221–10.850).

**Table 3:** Cox’s regression analysis demonstrating the prognostic potential of mutations identified in this study

Variable	Hazard ratio	95 % CI	P
Age of onset (<50 years old)	0.262	0.062–1.102	0.068
Lymph node metastasis	4.559	1.582–13.139	0.005
Tumor grade	1.093	0.424–2.820	0.854
PIK3CA mutations	1.421	0.282–7.163	0.670
KRAS mutations	1.635	0.493–5.414	0.421
EGFR mutations	3.639	1.221–10.850	0.020

## Discussion

We have identified TP53 in this study as the most commonly mutated gene in CRC from a group of 50 genes included in the cancer hotspot panel v2. Although expected, as TP53 is a tumor suppressor protein that is commonly mutated in many types of cancer, the increase from TP53 mutations frequency as reported by COSMIC database [12] is noteworthy. Mutant TP53 is an emerging target for cancer treatment using small molecule therapeutics that restores wild-type TP53 function in inducing cell cycle arrest and apoptosis. One of such molecules is the PRIMA-1/APR-246 small molecule which is showing promising results in phase I/II clinical trials [13].

The adenomatous polyposis coli (APC) gene was the second most commonly mutated gene in our cohort with 36.4 % of the cases examined displaying missense, nonsense or frameshift mutations in the hotspot regions of this gene. The most common mutation identified was the p.Arg1450Ter change resulting in the expression of truncated APC and thus loss of control on nuclear β-catenin mediated gene expression and dysregulation of the WNT pathway. APC mutations do not exhibit any significant prognostic value in our cohort although it has been shown previously that wild-type APC may confer a favorable prognosis in microsatellite stable CRC tumors only [14]. Mutations in the TGFβ pathway are represented by the alterations in SMAD4 in our cohort. Interestingly, we have detected pathogenic SMAD4 somatic missense variants previously reported in cases of juvenile polyposis syndrome [15] in 6 adult CRC patients. EGFR mutational rate detected in this study is higher than what is reported in the COSMIC database (11.1 % and 4 %, respectively). This relatively high mutation rate of EGFR in CRC may present itself as an opportunity for the use of the non-small cell lung carcinoma tyrosine kinase inhibitors (TKIs) treatment regimes targeting this receptor. This finding is of interest as it may influence the therapeutic outcome of chemotherapeutic drugs such as erlotinib or gefitinib [16]. PTEN is another gene that is mutated at a relatively high frequency in our cohort of CRC samples (13.1 %). PTEN functions as a tumor suppressor by negatively regulating AKT/PKB signaling pathway through the negative regulation of the intracellular levels of phosphatidylinositol-3,4,5-trisphosphate in cells. PIK3CA is the other frequently mutated gene in this pathway and it is significantly associated with poor disease-free survival.

## Conclusions

The frequent EGFR mutations identified in this cohort suggest an alternative therapeutic targeting avenue where lessons learnt from the treatment of lung cancer (the cancer type with the highest frequency of EGFR mutations detected) can be applied. In addition, high throughput targeted sequencing could reveal the interplay between different mutations and could elucidate their potential as prognostic markers as we show in this study for KRAS and PIK3CA mutations. Furthermore, understanding the molecular landscape of CRC in different populations will help in designing assays where the detection of frequently mutated genes will strongly indicate the presence of tumor growth, thus aiding easier diagnosis and large-scale screening programs.

## Abbreviations

CRC: colorectal cancer
CHP: cancer hotspots panel
KSA: Kingdom of Saudi Arabia
KAUH: King Abdulaziz University hospital
KFSHRC: King Faisal Specialist Hospital and Research Center
COSMIC: catalogue of somatic mutations in cancer
DSS: disease-specific survival
